# Supplementary material for: The effects of genital myiasis on the diversity of the vaginal microbiota in female Bactrian camels
Source: BMC Vet Res. 2022 Mar 5;18:87. doi: 10.1186/s12917-022-03189-5 (PMC8897907; doi:10.1186/s12917-022-03189-5)
Supplement: Supplementary file 5 — Additional file 5. [file 12917_2022_3189_MOESM5_ESM.zip › MPL201709200_16s_yy/Treat1/css/js_css/jsPDF-master.zip!/jsPDF-master/examples/html2pdf/showcase_supported_html.html]

HTML Test File


# HTML Test File

This page showcases all HTML supported features.

---

# Headings

# Heading 1

## Heading 2

### Heading 3

#### Heading 4

##### Heading 5

###### Heading 6

---

# Colors

Red Text With Green Background

---

# Font Sizes

12 Point

24 Point

---

# Fonts

Arial

Georgia

Terminal

Times

---

# Tables

|  |  |  |
| --- | --- | --- |
| Number | English | Spanish |
| 1 | one | uno |
| 2 | two | dos |
| 3 | three | tres |

# Images

Images are subject to CORS restrictions an may only show up in a production environment.
When testing, we start Chrome with the '--allow-file-access-from-files' flag...

# Lists

|  |  |  |
| --- | --- | --- |
| Default   - Milk - Eggs - Cheese   Circle   - Milk - Eggs - Cheese   Disk   - Milk - Eggs - Cheese   Square   - Milk - Eggs - Cheese | Default   1. Milk 2. Eggs 3. Cheese   Lower Alpha   1. Milk 2. Eggs 3. Cheese   Upper Alpha   1. Milk 2. Eggs 3. Cheese | Lower Roman   1. Milk 2. Eggs 3. Cheese   Upper Roman   1. Milk 2. Eggs 3. Cheese   Lower Greek   1. Milk 2. Eggs 3. Cheese   Upper Greek   1. Milk 2. Eggs 3. Cheese |

# Hyperlinks

## Internal

Colors

Font Sizes

Fonts

Tables

## External

www.twelvetone.tv
